# Solution-Processed Cu_2_Se Nanocrystal Films with Bulk-Like Thermoelectric Performance

**DOI:** 10.1038/s41598-017-02944-1

**Published:** 2017-06-05

**Authors:** Jason D. Forster, Jared J. Lynch, Nelson E. Coates, Jun Liu, Hyejin Jang, Edmond Zaia, Madeleine P. Gordon, Maxime Szybowski, Ayaskanta Sahu, David G. Cahill, Jeffrey J. Urban

**Affiliations:** 1 The Molecular Foundry, Materials Sciences Division, Lawrence Berkeley National Laboratory, 1 Cyclotron Road, Berkeley, California 94720 USA; 2 Nanosys, Inc., 233 South Hillview Drive, Milpitas, California 95035 USA; 3 California State University Maritime Academy, 200 Maritime Academy Drive, Vallejo, California 94590 USA; 4 North Carolina State University, Department of Mechanical and Aerospace Engineering, Raleigh, North Carolina 27695 USA; 5 University of Illinois at Urbana-Champaign, Department of Materials Science and Engineering, 1304 W. Green Street, Urbana, Illinois 61801 USA; 6 University of California, Department of Chemical Engineering, Berkeley, California 94720 USA; 7 UBS AG, Zurich, Switzerland

## Abstract

Thermoelectric power generation can play a key role in a sustainable energy future by converting waste heat from power plants and other industrial processes into usable electrical power. Current thermoelectric devices, however, require energy intensive manufacturing processes such as alloying and spark plasma sintering. Here, we describe the fabrication of a p-type thermoelectric material, copper selenide (Cu_2_Se), utilizing solution-processing and thermal annealing to produce a thin film that achieves a figure of merit, ZT, which is as high as its traditionally processed counterpart, a value of 0.14 at room temperature. This is the first report of a fully solution-processed nanomaterial achieving performance equivalent to its bulk form and represents a general strategy to reduce the energy required to manufacture advanced energy conversion and harvesting materials.

## Introduction

We are in urgent need of strategies for sustainable manufacturing, particularly for sectors related to energy production and storage^1^. The U.S. Department of Energy 2015 Quadrennial Technology Review (QTR)^2^ highlights a number of challenges to be overcome as well as approaches to be followed that will be necessary to bring about a more sustainable energy and manufacturing future. There are many approaches to minimizing the amount of energy and material wasted during manufacturing, including the reduction of processing temperatures as well as the number of heating steps, increased use of additive manufacturing techniques, production of combined heat and power systems, development of more efficient direct energy conversion materials, and deployment of widely distributed sensor networks for real time process monitoring and control. Thermoelectric power generators (TEGs) are solid-state, direct heat to electrical energy devices that can address the above issues in multiple ways. Firstly, the broad deployment of TEG-powered sensors in industrial facilities can provide more real-time process information. And secondly, improvements in the manufacture of TEGs themselves is necessary to facilitate their broader application.

Current state-of-the-art TEG devices are made of rare and/or toxic elements, such as tellurium, bismuth, and lead and are produced using wasteful and labor-intensive manufacturing techniques^2^. The composition and processing of the TEG material we describe in this work addresses both of these shortcomings. The constituent elements we use, copper and selenium, are relatively Earth-abundant and relatively non-toxic. The manufacture of current TEG components involves a series of energy-intensive processing steps including high temperature alloying, ball milling, and spark plasma sintering. For example, the production of bulk Cu_2_Se involves alloying the material at temperatures above 700 °C for more than a week and spark plasma sintering the material at a temperature above 400 °C while applying mechanical pressure of 65 MPa^3^. Once blocks of the material are produced, they are sawn into small pieces, resulting in a significant amount of kerf waste, and then assembled by hand into the final devices^2^. The nanocrystal solution we study lends itself to solution-processing based fabrication techniques and is therefore amenable to additive manufacturing processes which waste very little active material.

While the possibility of solution processed manufacturing of TEGs is exciting, one must not neglect the fundamental performance of the active materials. In the case of thermoelectric materials, material performance is most frequently assessed with the figure of merit, *ZT* = *S* ^2^ *σT*/*κ*, where *S* is the Seebeck coefficient, *σ* is the electrical conductivity, *T* is the absolute temperature, and *κ* is the thermal conductivity. As we can see from the *ZT* formula, an ideal material would be a poor heat conductor and a good electrical conductor, properties that are captured by the so-called “phonon glass, electron crystal” concept advanced by Slack and coworkers^4, 5^. One of the most confounding problems in maximizing *ZT* is the inherent coupling of thermal and electrical conductivities: materials with good electrical conductivity tend to have high thermal conductivity as well. Historically, a common strategy has been to engineer crystal domain sizes to scatter as many long mean free path (>100 nm) phonons^6^ without infringing on the transport of relatively short mean free path (<10 nm) charge carriers. Towards this end, researchers aiming to improve the thermoelectric performance of different materials incorporate nanoscale features such as grain boundaries to enhance scattering of long mean free path phonons without greatly perturbing the transport of shorter mean free path electrons^7–12^.

Since its emergence in the early 1990s, colloidal synthesis of nanocrystals has been regarded as a promising way to make device building blocks because of the exquisite control over composition, size, shape, and crystallographic phase it provides^13^. Indeed, the proliferation of recipes for metallic and semiconducting nanocrystals we have witnessed over the past few decades has enabled the solution-processing based manufacture of a wide variety of inorganic and inorganic/organic hybrid materials^10^. Examples in the literature are abundant and include reports of photovoltaics^14–16^ and other optoelectronic devices^17, 18^, electronic devices such as field-effect transistors^19–22^, and, of particular relevance to this work, thermoelectrics^8, 12, 23–28^.

In addition to potential economic and manufacturing efficiency gains, solution processing lends itself to the production of devices that are of arbitrary geometry and mechanically flexible. In the case of thermoelectrics, novel geometries and flexibility will broaden the horizon for the application of thermoelectric power generators, assuming efficient heat exchangers can be applied^2, 8^. An already emerging market of devices that utilize TEGs is body-powered electronics such as watches, as well as devices for medical monitoring and diagnostics^29, 30^. While traditionally manufactured TEGs are generally rigid and flat, some efforts have been made to manufacture flexible devices using pixelated, paneled devices, but at the expense of several additional processing steps^31, 32^. The direct, bottom-up production of TEGs that solution processing enables is actively being developed in our and other groups^22, 23, 33–35^.

In this manuscript, we describe the synthesis and thermoelectric properties of copper selenide (Cu_2_Se) nanocrystals which combine the advantages of solution processing with the benefits of an inherently nanostructured material. Ultimately, we demonstrate that solution-processed thin films of these nanocrystals achieve a material *ZT* of 0.14 at room temperature, which is as high as their bulk counterparts^3^, which also have a *ZT* of 0.14 at 290 K, while exploiting solution processing and mild thermal annealing.

## Results

### Synthesis of Cu_2_Se Nanocrystals

Cu_2_Se nanocrystals were synthesized via a modified version of a previously published protocol^36^; the details of our protocol are described in the methods section. The product of our synthesis is a suspension of oleylamine-coordinated nanocrystals in hexane. The Cu_2_Se nanocrystals have an average diameter of 12 +/− 1 nm, as determined from analysis of low-resolution TEM images.

**Figure 1:**
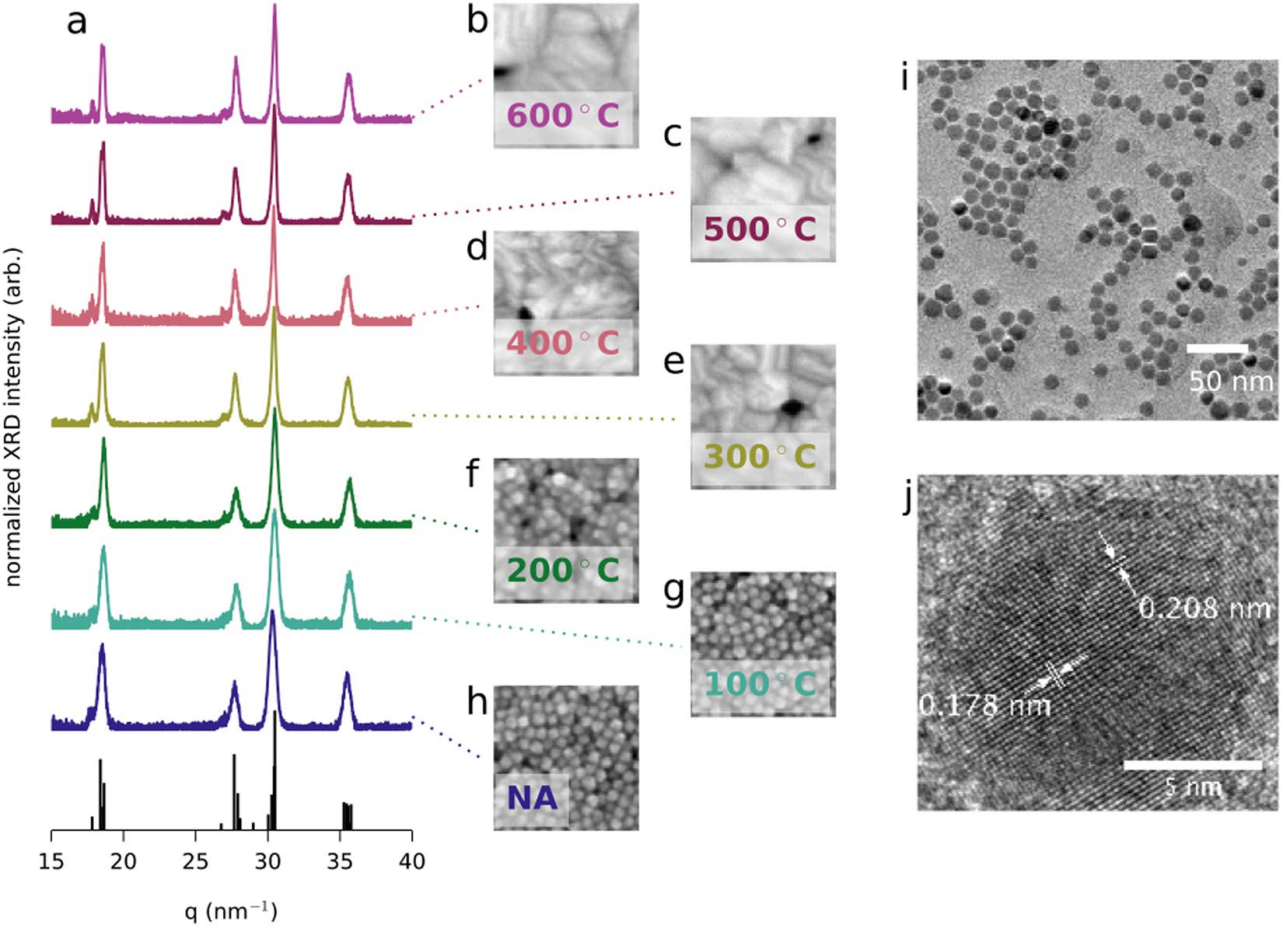
Nanocrystal structural characterization. (**a**) XRD spectra for drop-cast and annealed films of Cu_2_Se. In this plot, *q* is in units of 2*π*/*d*. The reference peaks are calculated from the room temperature structure determined by Gulay *et al*.^37^. (**b–h**) Top-view SEM images of thin films that were annealed at the same temperature as the drop-cast samples used for XRD spectra in (**a**). In panel (**h**), “NA” stands for “not annealed”. Each image is 170 nm across. (**i**) Low resolution TEM image of Cu_2_Se nanocrystals. (**j**) High resolution TEM image of a Cu_2_Se nanocrystal.

The particles themselves are crystalline, as evidenced by the lattice fringes visible under high-resolution TEM imaging (see Fig. 1j). While we refer to our copper selenide particles as stoichiometric (Cu_2_Se), copper selenide actually has a stable monoclinic structure in compositions ranging from Cu_2_Se to Cu_1.8_Se. We are confident that our material is in the monoclinic phase because the XRD pattern matches the monoclinic structure determined by Gulay *et al*. (see Fig. 1a)^37^. Furthermore, by comparing the room temperature Hall carrier concentration of our film annealed at 300 °C, *n*_*Hall*_ ≈ 9 × 10^20^ cm^−3^ (see Supplementary Figure 1), to data in recent work by Kang *et al*.^38^, we estimate that our material has a composition of Cu_1.94_Se, assuming that the concentration of holes is proportional to degree of copper deficiency and that the hole mobility is constant in this range of compositions. In the end, we do not know the precise stoichiometry of our material and will refer to it as Cu_2_Se in this manuscript.

### Thin Film Preparation and Characterization

**Figure 2:**
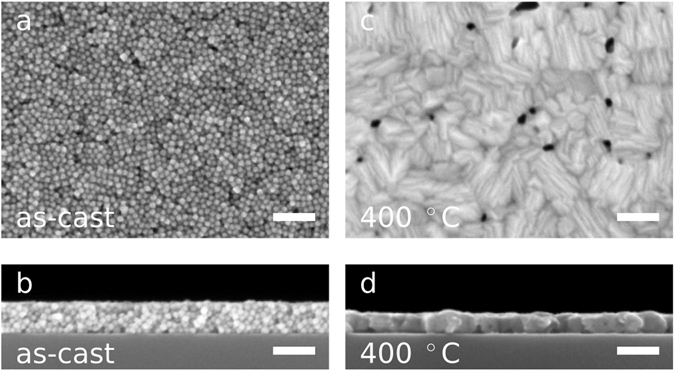
Thin Film Morphology. (**a**) Top-view SEM image of an as-cast thin film of Cu_2_Se nanoparticles. (**b**) Cross-section SEM image of the film shown in (**a**). The film is 75 nm thick. (**c**) Top-view SEM image of a Cu_2_Se thin film after annealing at 400 °C for 45 minutes in an N_2_ atmosphere. Prior to annealing, this film was prepared the same way as the film in (**a**). (**d**) Cross-section SEM image of the film shown in (**c**). The film is 54 nm thick. The scale bars in all images are 100 nm wide. The full sets of top-view and cross-section SEM images are shown in Supplementary Figures 2 and 3.

In order to assess Cu_2_Se nanocrystals as a thermoelectric material, we spin cast them from a suspension in a mixture of hexane and octane *via* a multi-step deposition procedure to fabricate uniform and crack-free films that are between 50 and 100 nm thick (see Fig. 2, panels a and b). Our use of a multi-step spin casting procedure is inspired by a technique which has been used to create dense films of lead selenide nanocrystals for photovoltaic applications^20^.

We then thermally anneal the films for 45 minutes under a N_2_ atmosphere at a temperature between 100 °C and 600 °C. After annealing, the films are generally reduced in thickness, but remain crack-free (see Fig. 2, panels c and d and Supplementary Figures 2 and 3). Annealing at temperatures up to 200 °C appears to densify the films, but the individual nanocrystals remain visible in the SEM, as can be seen in Fig. 1, panels f-g and in Supplementary Figures 2 and 3. After annealing at temperatures of 300 °C and above, the individual nanocrystals are no longer distinct, as can be seen in the SEM images Fig. 1, panels b-e, and confirmed by the disappearance of the form factor peaks in the low *q* region of GIWAXS patterns (Supplementary Figure 4). Transmission FTIR spectra shown in Supplementary Figure 13 indicate a significant amount of EDT in the not annealed films and a significant loss of EDT after annealing at 200 °C. Samples annealed at even higher temperatures, 400 °C and 600 °C, show no absorption from EDT, indicating that the ligands have been effectively removed at these elevated annealing temperatures.

While the morphology of the films clearly changes during the annealing procedure, the room temperature crystallographic structure of the material remains consistent through the entire range of annealing temperatures used in this work. The powder XRD spectra shown in Fig. 1a were recorded from drop-cast and annealed samples because the thin films do not scatter strongly enough to get acceptable signal-to-noise ratios on our XRD system. GIWAXS patterns, which were obtained using synchrotron X-rays, confirm that the crystallographic structure of our thin films is maintained throughout the annealing series (see Supplementary Figure 4). The individual nanocrystals appear to merge and grow into larger crystallites with higher annealing temperature, but we cannot estimate the crystallite size from our XRD data because reflections from multiple lattice planes contribute to many of the peaks, as can be seen in Fig. 1a.

### Thermoelectric Performance

**Figure 3:**
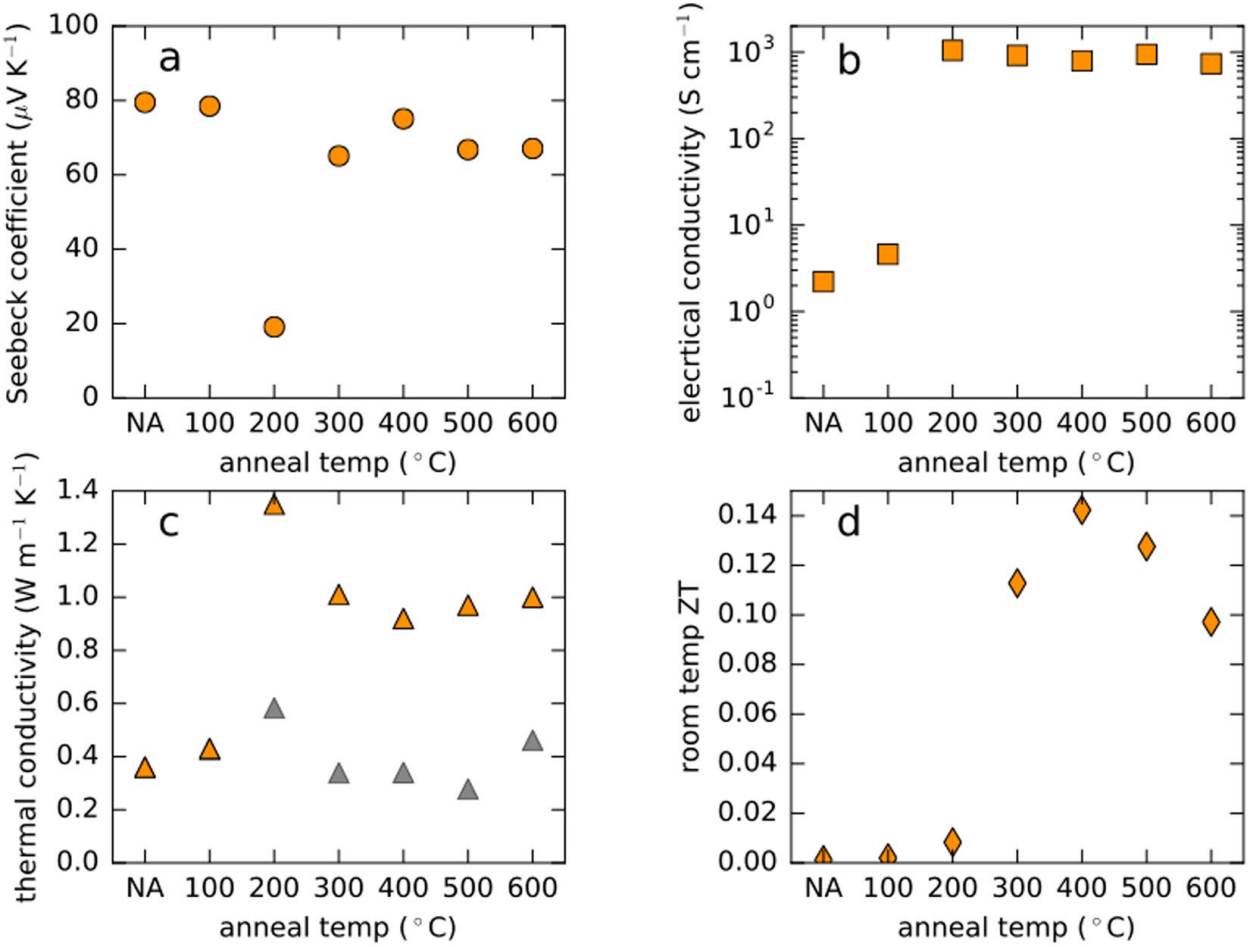
Thermoelectric Performance as a Function of Annealing Temperature. (**a–d**) Room temperature values for Seebeck coefficient, in-plane electrical conductivity, cross-plane thermal conductivity, and *ZT*, respectively, plotted as functions of annealing temperature (“NA” stands for “not annealed”). The gray symbols in panel (**c**) are estimates of the lattice thermal conductivity, which are computed using the Wiedemann-Franz Law, *κ*_*lattice*_ = *κ*_*total*_ − *σ*_*e*_ *LT*, where *κ*_*total*_ = *κ*_*electronic*_ + *κ*_*lattice*_ is the total thermal conductivity measured by TDTR, *σ*_*e*_ is the electrical conductivity of the film, *L* = 2.44 × 10^−8^ *W* Ω *K* ^−2^ is the Lorenz number in the degenerate limit, and *T* is room temperature in Kelvins. The data in this figure are from samples derived from the same synthetic batch. We report the results, except for thermal conductivity, for a second batch of nanocrystals in Supplementary Figure 12.

The apparent changes in the structure of the films correlate with changes in the measured thermoelectric properties (Fig. 3). The non-annealed and 100 °C annealed samples both have Seebeck coefficients of approximately 80 µV K^−1^ and have electrical conductivities below 10 S cm^−1^. The sample annealed at the next higher temperature, 200 °C, displays a roughly 75% drop in the magnitude of the Seebeck coefficient and a greater than two order of magnitude increase in electrical conductivity. This sample is also the lowest annealing temperature sample to lose the form factor fringes in GIWAXS (see Supplementary Figure 4), implying that the material contrast of the particle-particle interfaces has been dramatically reduced.

At the same time as the thermopower and the electrical conductivity, both measured in-plane, are undergoing significant changes, the cross-plane thermal conductivity as measured by time domain thermal reflectance (TDTR) is also changing (Fig. 3c). Annealing the sample at 200 °C results in its thermal conductivity increasing roughly 250%, from 0.4 W m^−1^ K^−1^ to 1.4 W m^−1^ K^−1^, relative to the 100 °C sample. Interestingly, the thermal conductivity drops as the annealing temperature is raised to 300 °C and beyond. Determining the cause of the behavior of the sample annealed at 200 °C is beyond the scope of this manuscript, but we emphasize that the trends in Fig. 3 are characteristic of films made from Cu_2_Se nanocrystals, as shown in Supplementary Figure 12. We believe that combining in-plane (Seebeck and electrical conductivity) and cross-plane (thermal conductivity) measurements to compute ZT is valid in this case because our material is built of randomly oriented spherical particles. Ultimately, taking the annealing temperature evolution of the transport parameters into account and computing ZT for our material reveals an optimal annealing temperature of 400 °C for room temperature performance, with a ZT of 0.14 (Fig. 3d).

## Discussion

We have demonstrated that mild thermal annealing can transform a film of isolated nanocrystals into a continuous thin film with thermoelectric properties rivaling those of intensively-processed bulk materials of the same composition. To our knowledge this is the first report of the complete characterization of a fully solution-processed nanocrystalline material that achieves a *ZT*, 0.14 in this case, as high as its bulk counterpart. These results prove the viability of nanocrystal-based inks as a component of solution-processed, additively-manufactured thermoelectric devices. The further development of this and similar materials will drive the efficient and economical manufacturing of energy harvesting devices which will in turn increase the efficiency of industrial processes.

## Methods

### Nanocrystal Synthesis

Cu_2_Se nanocrystals were synthesized following a slightly modified previously published synthesis^36^. A solution of copper(I) chloride in oleylamine (OAm) and 1-octadecene (ODE) at 110 °C was injected into a solution of selenium dissolved in the same solvents at a temperature of 310 °C. The reaction was allowed to proceed for 20 minutes at 300 °C. The 12 +/− 1 nm diameter nanocrystals were washed by precipitating with ethanol and resuspended in hexane twice. The nanocrystals were kept in hexane and stored in a N_2_ glove box for future use.

### Thin Film Deposition, Ligand Exchange, and Annealing

Thin films of Cu_2_Se nanocrystals were prepared by spin casting onto clean, 1 cm × 1 cm substrates (doped silicon for SEM imaging and thermal conductivity or glass for DC conductivity and thermopower) from a 3:2 volume ratio mixture of hexane and octane. Films were produced by pipetting 30 µl of a dilute suspension (~15 mg/ml) of nanocrystals and spinning at 2000 rpm for 30 seconds, resulting in an incomplete monolayer. Ligand-exchanged thin films of Cu_2_Se were prepared by repeated spin coating of sub-monolayers of OAm-coordinated nanocrystals from a solution of hexane and octane. After each deposition of OAm-coordinated NCs, 60 µl of an ethanedithiol (EDT) solution (1% by volume in acetonitrile, ACN) was pipetted onto the film. The EDT solution was left in contact with the film for approximately 30 seconds while the original OAm ligands were displaced by the EDT ligands. After the ligand exchange step, the film was spun to remove the excess solution and was rinsed once with neat ACN. As a result of the ligand exchange, the nanocrystals in the film were no longer soluble in the original non-polar solvents, allowing for multiple depositions of OAm-coordinated NCs to reach the target thickness. After the target thickness was achieved, the films were annealed at the specified temperature for 45 minutes in a nitrogen glove box.

### XRD Analysis

Powder XRD patterns were recorded using a Bruker AXS D8 Discover GADDS X-Ray Diffractometer using Co K *α* radiation (*λ* = 1.789 Å). The reference pattern was calculated with *CrystalMaker* (CrystalMaker Software Limited) software using the structural parameters from ref. 37.

### SEM and TEM Imaging

SEM images were obtained with a Zeiss Gemini Ultra-55 Analytical Field Emission Scanning Electron Microscope operated at 5 kV using an in-lens detector. TEM images were recorded using a JEOL 2100-F Field-Emission Analytical TEM at 200 kV. TEM samples were prepared by placing a single drop of dilute nanocrystal solution onto a TEM grid (400-mesh Cu on ultrathin carbon – Ted Pella 01822-F) and the hexane was allowed to evaporate. Image-J software was used to analyze TEM images to determine the particle size distribution (n = 76).

### FTIR Transmission Spectra

FTIR transmission spectra were collected using a Perkin Elmer Spectrum One FT-IR system for a set of four EDT-exchanged films: not annealed, and annealed at 200 °C, 400 °C, and 600 °C. These films were produced in the same manner as the other samples in this study with the exception of the use of a different substrate. Samples for FTIR were spin cast onto undoped double-side polished silicon to allow for collection of spectra in transmission mode. The films were annealed in a nitrogen glovebox. The spectra were recorded in air immediately after removing them from the glovebox.

### Thermoelectric Transport Characterization

Before performing electrical and thermoelectric measurements, 100 nm thick gold contacts were thermally evaporated onto each of the four corners of the thin film samples. Sheet resistance for each film was measured in a 4-point Van der Pauw configuration with two Keithley 2400 sourcemeters. Electrical conductivity was determined from the sheet resistance and the thickness for each film. Film thickness was measured from cross section SEM images like those shown in Fig. 2, panels b and d, and in Supplementary Figure 3. Thermopower (Seebeck coefficient) was measured using a homemade probe station which features two Peltier devices (Ferrotec) placed approximately 4 mm apart with a single current passed through the devices in opposite polarities. As a result, one device heats and the other device cools in approximately the same amount relative to room temperature. A thermal gradient was induced in the sample by laying the thin film across the gap between the two devices. Thermal paste (Wakefield Thermal S3 Solutions) was used to ensure good thermal contact. The open circuit voltage that developed was measured using an Agilent 34401 multimeter. The thermal gradient in the sample was monitored with two T-type thermocouples mounted in micromanipulators. For each sample, five different temperature gradients were established and allowed to equilibrate for 200 seconds between temperature changes with 10 voltage measurements taken and averaged at each temperature difference. All samples showed a linear dependence of open circuit voltage on the magnitude of the temperature gradient; this trend was used to extract thermopower values. Homemade LabVIEW programs were used to collect both electrical conductivity and thermopower data. Our Seebeck measurement procedure was verified by measuring the Seebeck coefficient of thermally evaporated nickel films on glass substrates.

### Thermal Conductivity Characterization

We measured the cross-plane thermal conductivity of the Cu_2_Se samples by the time-domain thermoreflectance (TDTR) method^39, 40^. A mode-locked Ti:Sapphire laser generated a train of pulses at 80 MHz with wavelength centered at 783 nm. The pulses were split into pump and probe beams traveling along different optical paths. The pump beam was modulated at 9.2 MHz by an electro-optic modulator and induced heat excursion on the sample surface, which was interrogated by the time-delayed probe beam. The 1/e^2^ radius of the focused beam on the sample surface was about 10 μm. Nb_0.43_V_0.57_ alloy with thickness around 70 nm was deposited as an optical transducer on the samples by magnetron sputtering. Prior to depositing the optical transducer, the films were coated with 5 nm of Al_2_O_3_ via room temperature ALD to protect the samples against oxidation and to physically isolate the nanocrystal film from the transducer layer. The reflected probe beam at the sample surface was detected by a Si photodiode connected to a radio-frequency lock-in amplifier referenced to 9.2 MHz. We compared the ratio of the in-phase voltages to the out-of-phase voltages across time delay with modeling to find the best fit for the thermal conductivity of Cu_2_Se and the interface thermal conductance. We use the Dulong-Petit limit of heat capacity for Cu_2_Se, 2.51 J cm^−3^ K^−1^, derived from its theoretical molar volume^41^, in the thermal model. See a summary of the raw data in Supplementary Figures 5–11.

### Data Availability

The datasets generated during and/or analysed during the current study are available from the corresponding author on reasonable request.

## Electronic supplementary material

Supplementary Information
