# Supplementary material for: Solution-Processed Cu2Se Nanocrystal Films with Bulk-Like Thermoelectric Performance
Source: Sci Rep. 2017 Jun 5;7:2765. doi: 10.1038/s41598-017-02944-1 (PMC5459815; doi:10.1038/s41598-017-02944-1)
Supplement: Supplementary file 1 — Supplementary Information [file 41598_2017_2944_MOESM1_ESM.pdf]

Supplemental Information for "Solution-Processed Cu_2Se Nanocrystal Films with Bulk-Like Thermoelectric Performance"

Jason D. Forster, Jared Lynch, Nelson E. Coates, Jun Liu, Hyejin Jang, Edmond W. Zaia, Madeleine P. Gordon, Maxime Szybowski, Ayaskanta Sahu, David G. Cahill, and Jeffrey J. Urban

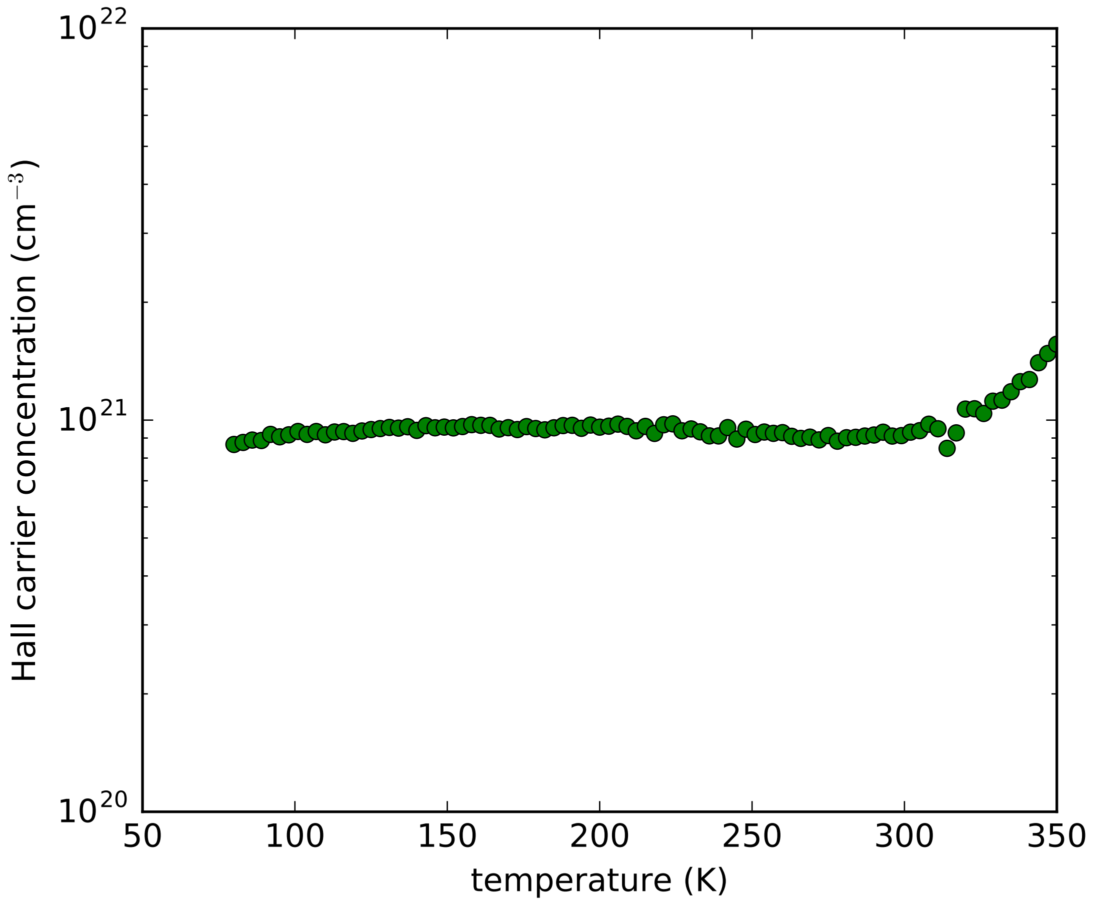

Supplementary Figure 1 | Temperature dependent Hall carrier concentration. Hall carrier concentration for the sample annealed at 300 °C. This measurement was carried out on an Ecopia HMS-5000 Variable Temperature Hall Effect Measurement System with an ~0.5 T magnet.

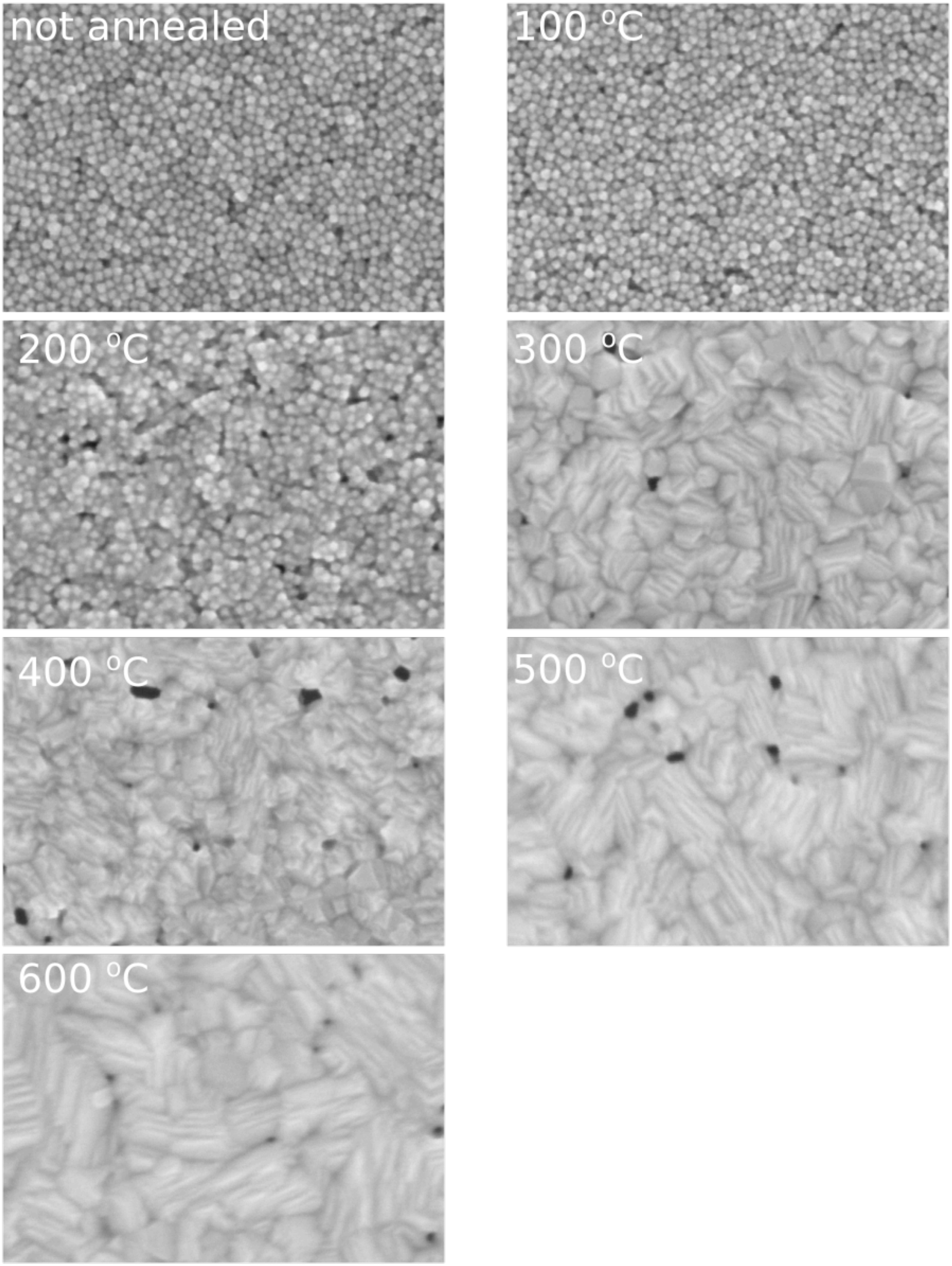

Supplementary Figure 2 | Top-view SEM Images. SEM images show the evolution of film morphology as a function of annealing temperature. Each is labeled with the annealing temperature and is 625 nm across.

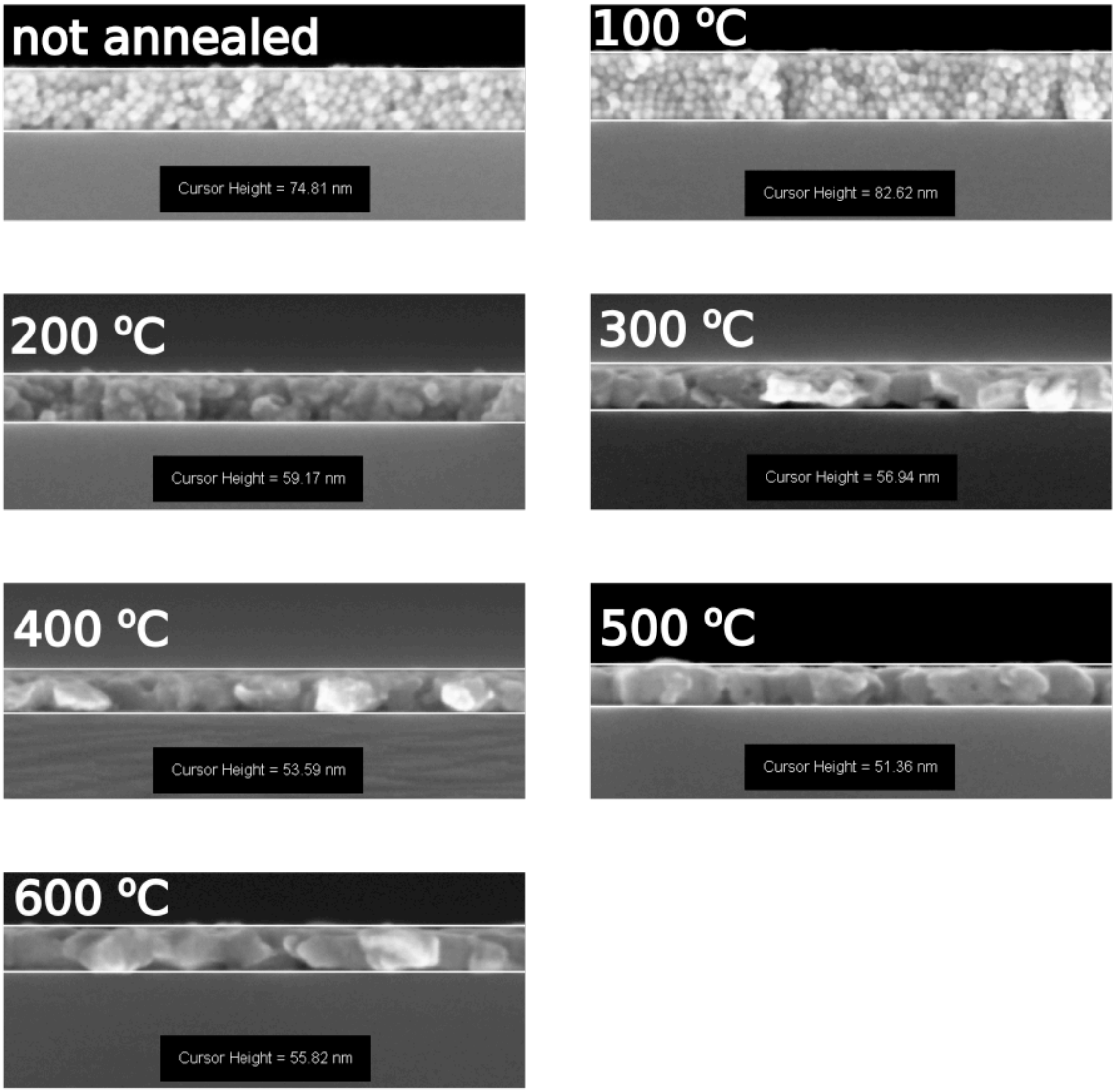

Supplementary Figure 3 | Cross-Section SEM Images. SEM images of Cu_2Se thin films that were used to determine film thicknesses. Each image is labeled with the annealing temperature and is 625 nm across. The thickness of each film is also indicated.

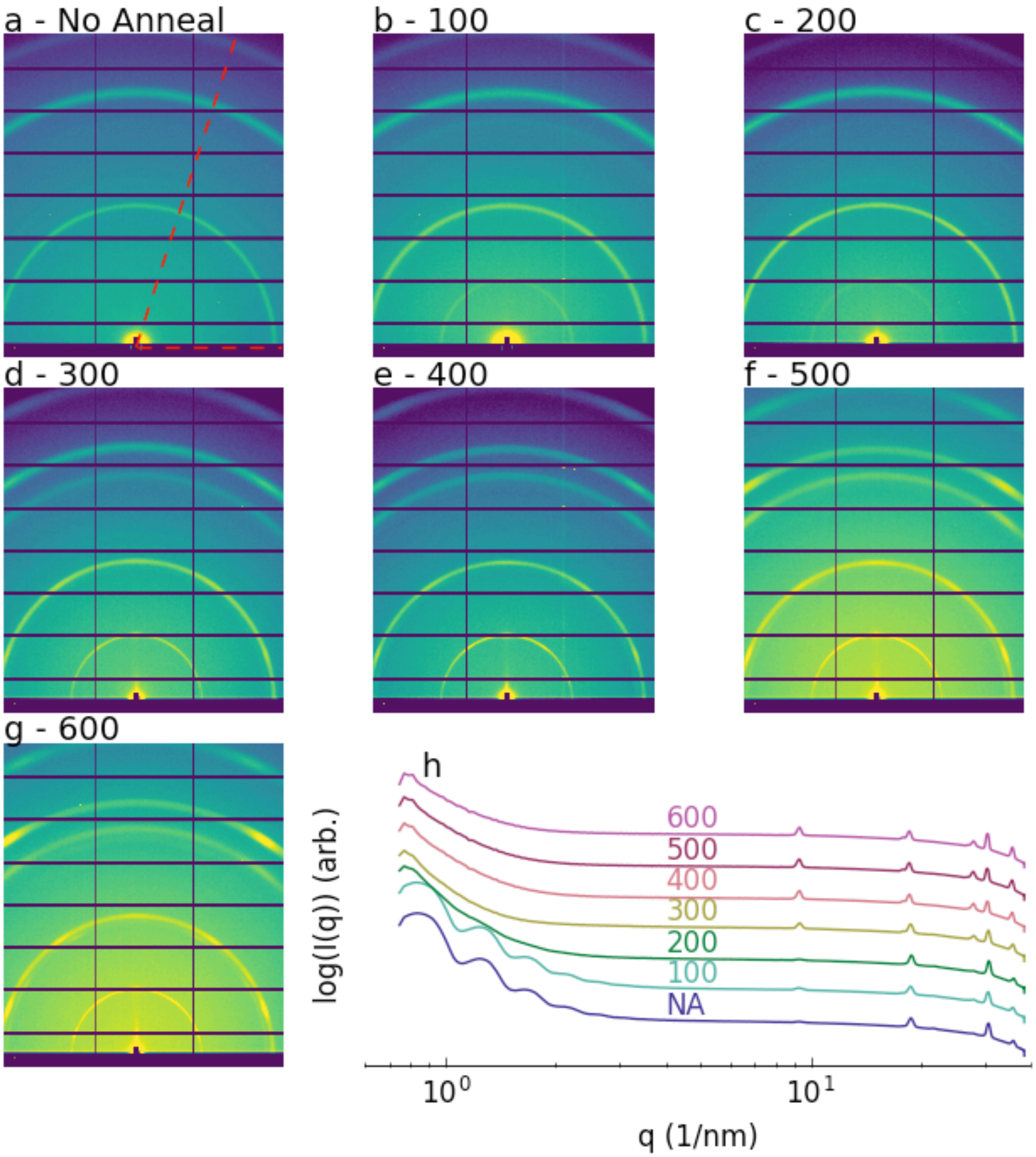

Supplementary Figure 4 | GIWAXS and azimuthal averages reveal structural evolution with annealing. Panels (**a-g**) show the logarithm of the scattered X-ray intensity for thin film samples for the full annealing series. Each image is 32.3 nm^{-1} wide in *q*-space and is labeled with the annealing temperature in degrees Celsius. (**h**) Azimuthal averages of the scattered X-ray intensity for each sample in the sector between the dashed red lines shown in (a).

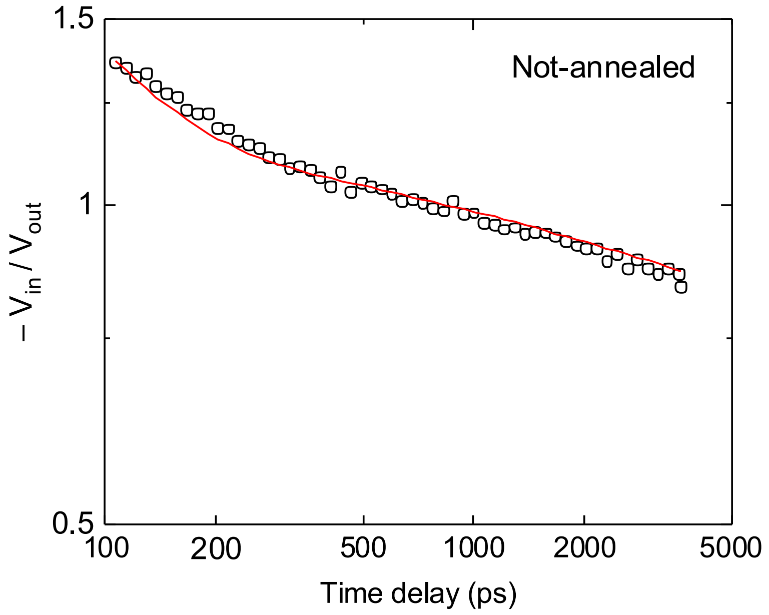

Supplementary Figure 5 | Not Annealed Time Domain Thermoreflectance Results. Ratio of in-phase to out-of-phase voltage response as a function of time delay.

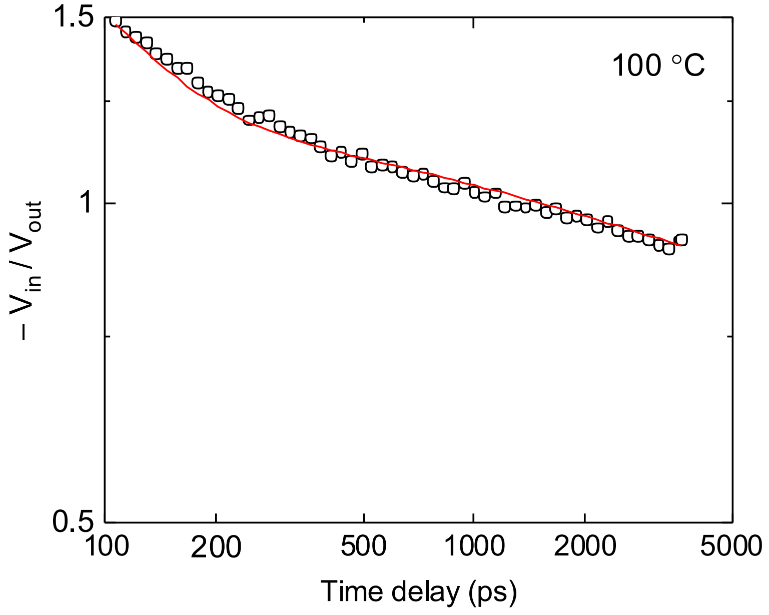

Supplementary Figure 6 | 100 °C Annealed Time Domain Thermoreflectance Results. Ratio of in-phase to out-of-phase voltage response as a function of time delay.

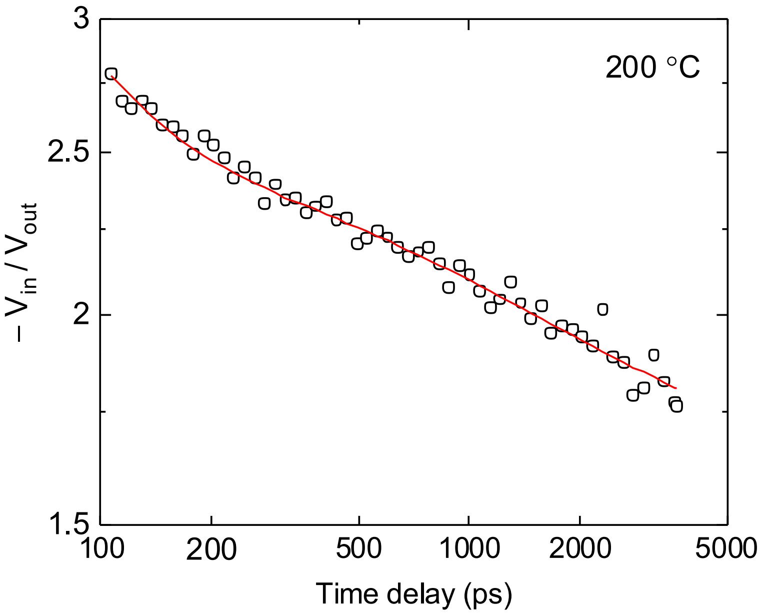

Supplementary Figure 7 | 200 °C Annealed Time Domain Thermoreflectance Results. Ratio of in-phase to out-of-phase voltage response as a function of time delay.

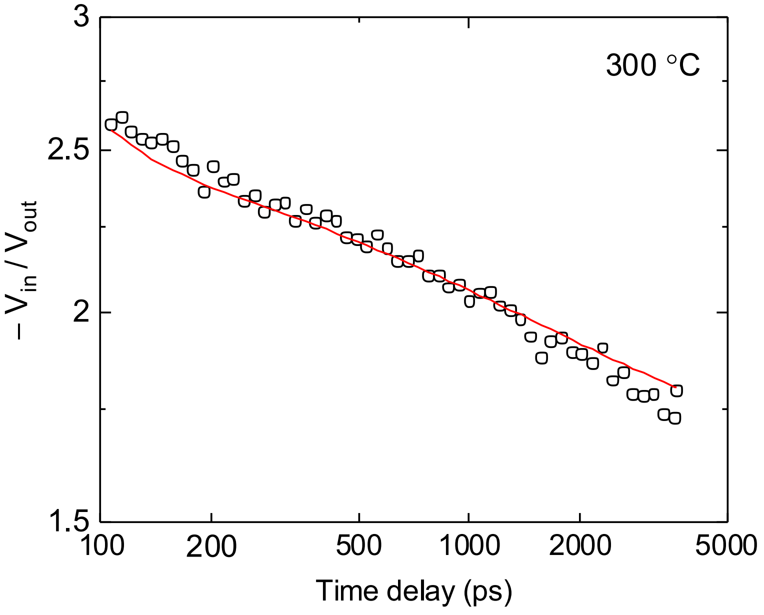

Supplementary Figure 8 | 300 °C Annealed Time Domain Thermoreflectance Results. Ratio of in-phase to out-of-phase voltage response as a function of time delay.

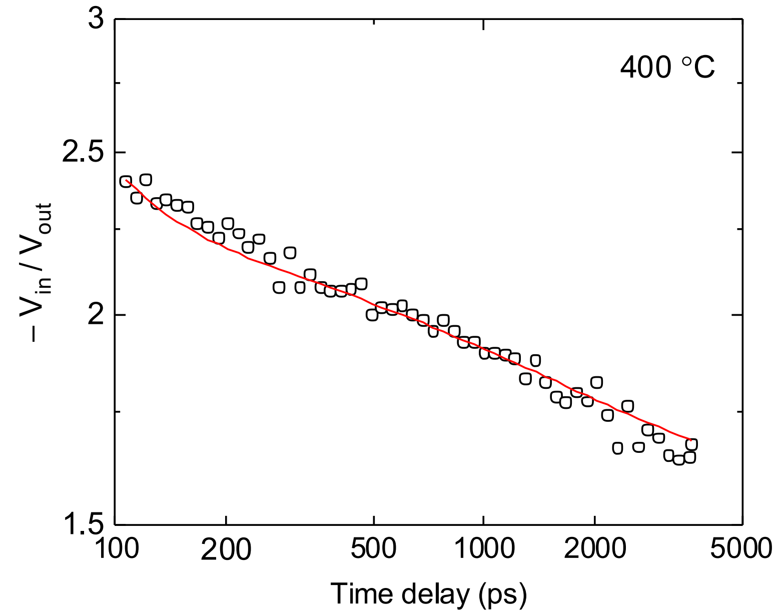

Supplementary Figure 9 | 400 °C Annealed Time Domain Thermoreflectance Results. Ratio of in-phase to out-of-phase voltage response as a function of time delay.

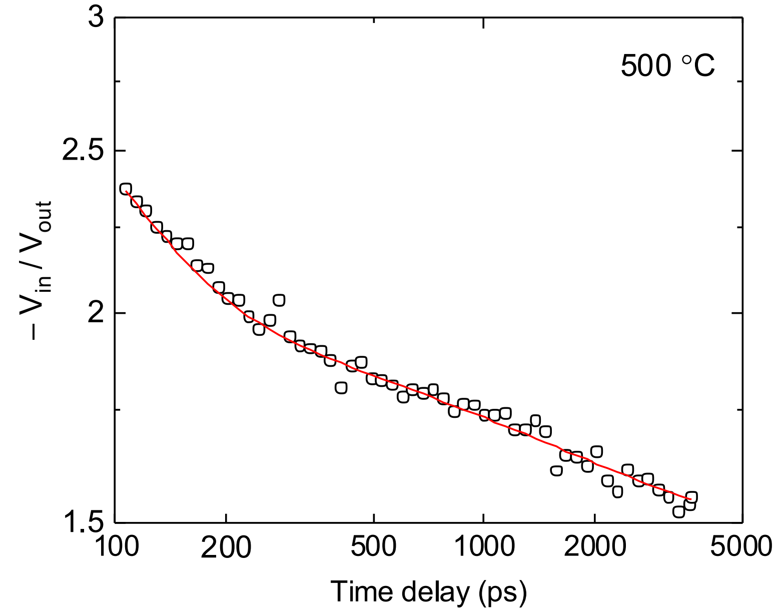

Supplementary Figure 10 | 500 °C Annealed Time Domain Thermoreflectance Results. Ratio of in-phase to out-of-phase voltage response as a function of time delay.

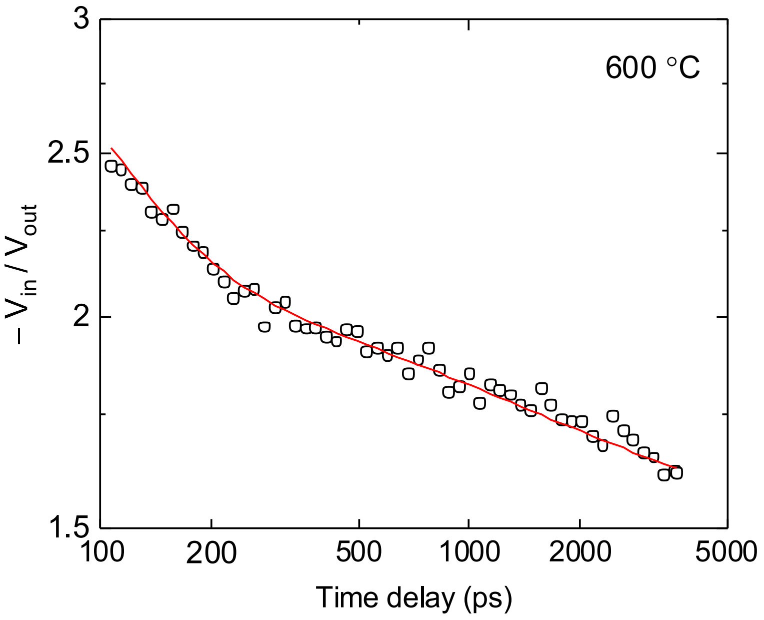

Supplementary Figure 11 | 600 °C Annealed Time Domain Thermoreflectance Results. Ratio of in-phase to out-of-phase voltage response as a function of time delay.

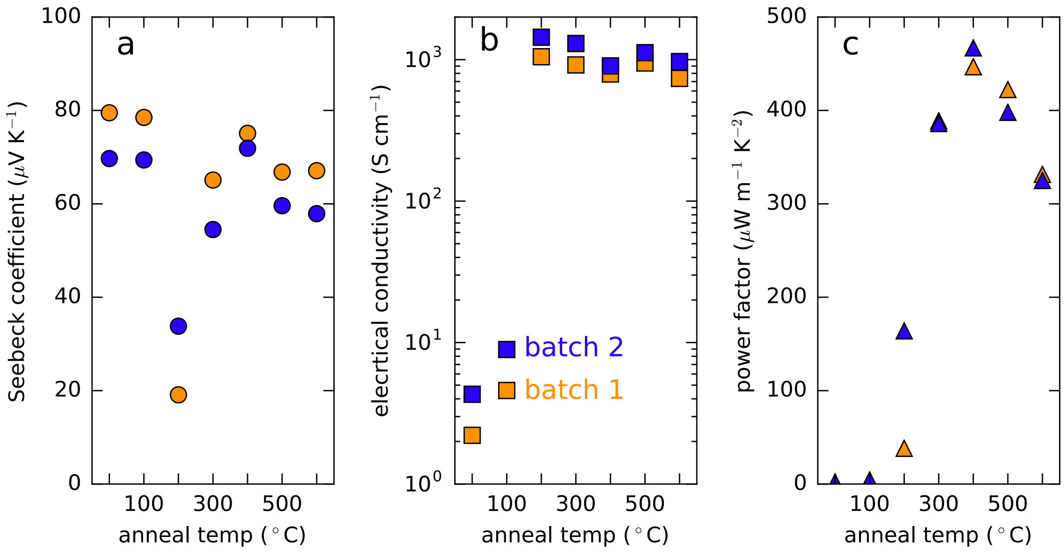

Supplementary Figure 12 | Thermoelectric Performance for Two Different Batches as a Function of Annealing Temperature. (**a-c**)Room temperature values for Seebeck coefficient, electrical conductivity, and power factor ($S^2\sigma$), respectively, plotted as functions of annealing temperature. The symbols in orange (batch 1) are the same data from the main text. The symbols in blue (batch 2) are from a set of films fabricated from a different synthetic batch of particles. Note that while the exact values for the films of each batch are different, the trends as functions of annealing temperature are repeatable. The differences in the specific values are may be attributable to small differences in the stoichiometry of the particles from batch to batch.

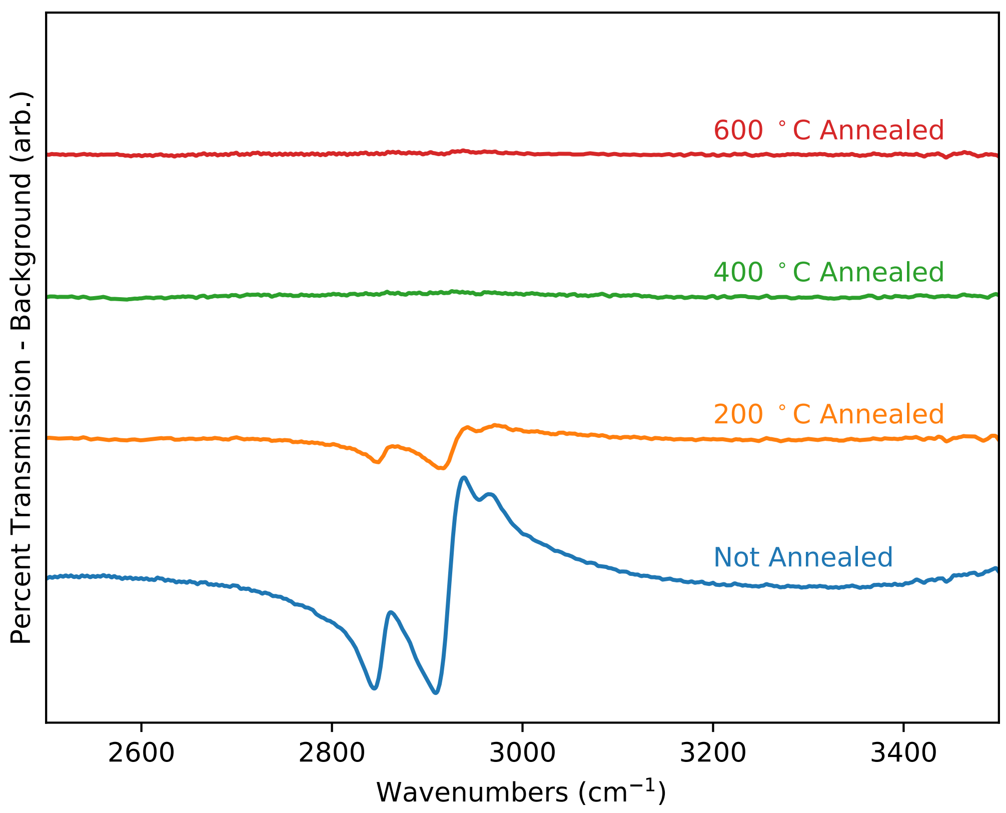

Supplementary Figure 13 | FTIR Spectra of Ligand-Exchanged Cu_2Se Nanocrystal Films. Background-corrected FTIR transmission spectra of four Cu_2Se nanocrystal films indicate the loss of EDT ligands during annealing at higher temperatures. The spectrum for the not annealed sample displays relatively strong absorption in the 2800 – 3000 cm^{-1} region of the spectrum, corresponding to the symmetric and asymmetric stretching modes of the C-H bonds in EDT. The sample annealed at 200 ^{o}C displays some absorption in this region, but the intensity is diminished compared to the not annealed sample. The samples annealed at 400 and 600 ^{o}C do not display any significant absorption in this region, indicating that the EDT ligands have been effectively removed. The intensity scale of these spectra is the same and they have been offset for clarity.
